# Adaptation of the Exercise Identity Measurement Scale to Turkish: a study of validity and reliability

**DOI:** 10.3389/fpsyg.2026.1741029

**Published:** 2026-03-18

**Authors:** Hulusi Mehmet Tunçkol

**Affiliations:** Necat Hepkon Faculty of Sport, Dokuz Eylül University, İzmir, Türkiye

**Keywords:** exercise, identity, reliability, scale, validity

## Abstract

Exercise is an important issue for modern societies. It should be a part of every human being’s life. People create an exercise identity based on their exercise habits. At this point, a measurement tool that determines the exercise identity of individuals is helpful for scientists. Therefore, this study aimed to assess the reliability and validity of the “Exercise Identity Measurement Scale” (EIMS) for the Turkish sample. The scale adaptation process involved collecting responses from 689 participants via a digital platform. The original “Exercise Identity Measurement Scale” (EIMS) is a nine-item inventory. Participants were asked to indicate the degree to which each statement was characteristic of them on a 7-point Likert-type scale. According to the results of this study, the eight-item unidimensional structure of the EIMS was found to be reliable and valid for the Turkish population.

## 1 Introduction

The public is increasingly engaging in regular exercise, and both laypeople and researchers are becoming more interested in studying exercise. The Exercise Identity Measurement Scale (EIMS) is a reliable and valid instrument in English-speaking societies. However, there is limited research examining the generalizability of its items and factor structure in non-English-speaking populations. During the scale adaptation process, conducting various analyses is essential to ensure the statistical validity of the scale.

As vital components of an individual’s self-concept, role identities help individuals in attributing significance and value to their past actions and provide direction for future behaviors. When role identities promote or motivate actions that are consistent with the identity, the prominence of the role identity can be seen as a significant predictor of behavior. However, due to the reciprocal nature of this relationship, engaging in some level of physical activity is necessary for the development of an exercise identity. Given that limited research has been conducted to explore the application of this perspective to behavior, further studies are needed to develop valid and reliable measures of the prominence of exercise identity (Anderson and Cychosz, 1994).

In the field of physical activity, the theoretical foundations of identity theory are indeed applicable to the role of the individual who exercises. Consequently, exercise identity not only impacts the perception of exercise-related stimuli but also provides guidance for future exercise actions. Identity theory suggests that the likelihood of activating one’s exercise identity as a basis for engaging in role-related behaviors (e.g., hiking on a sunny day) reflects the position of exercise identity within the individual’s identity salience hierarchy (Liardi, 2015). Liardi (2015) stated that the more individuals regard exercise as a vital aspect of their identity, especially in relation to other potential roles, the more likely it is to occupy a prominent position within their identity hierarchy. This enhances the probability of engaging in exercise behaviors that serve to reaffirm this identity.

When reflecting on what it means to be an individual who exercises, people may refer to and endorse shared societal interpretations of this role, such as the belief that “exercisers participate in frequent and consistent physical activity” (Strachan et al., 2017). Anderson and Cychosz's (1994) conceptualization of exercise identity proposed that has a strong exercise identity assigns significant value to their participation in sports and has an awareness of their self-perceptions within the exercise domain.

Behavior patterns are essential to the development of role identities. As individuals engage in activities associated with their role identities or enact various aspects of the exerciser role, they may, through social interaction, have their identity as an exerciser reinforced and validated. Simultaneously, this validation of the role identity increases the likelihood of exercise-related actions in the future. Once behaviors associated with the role identity, such as being an exerciser, are initiated and integrated into one’s self-concept, they are likely to develop into primary salient beliefs. If, through behavioral participation and social interaction, the exerciser role identity becomes a valued component of one’s self-concept and a primary salient belief, it may also play a critical role in guiding future exercise actions (Anderson et al., 2001). One notion that may be appealing in this context is the idea of identity (Wilson and Muon, 2008). Therefore, possessing a strongly endorsed exercise identity may promote identity-consistent actions (e.g., exercise) (Strachan et al., 2009). Identity-relevant concepts are increasingly acknowledged in behavioral theories and research, and a related branch of identity theory also recognizes the importance of identity in shaping behavior (Alfrey et al., 2023).

The substantial evidence linking exercise identity with measures of exercise behavior, combined with the limited research on the role of this construct in the motivational processes that drive exercise participation, justifies the exploration of the exercise identity construct (Vlachopoulos et al., 2011). Merely understanding the benefits of regular exercise and lifelong physical activity for health is insufficient to strengthen an individuals’ exercise identity (Soukup Sr et al., 2010). Individuals who exercise regularly repeatedly experience success in self-regulation, thereby strengthening their self-regulation abilities with each completed workout. In contrast, those who exercise occasionally, by definition, rarely experience this kind of success and may even feel a sense of failure when they realize they are not adhering to their exercise routines (Iso-Ahola, 2013). Therefore, having a strong exercise identity can help with the self-regulation of exercise (Strachan et al., 2013).

Several factors influence the development of exercise-related cognitions. Various elements contribute to the formation of an exercise identity, including feelings of achievement, autonomy, control, social interaction, and a sense of belonging (Hardcastle and Taylor, 2005). ‘Exercise identity’ is consistently associated with exercise behavior and affective and social-cognitive variables, all of which play a critical role in regulating exercise (Berry et al., 2013). It is necessary to implement intervention programs specifically designed to target mechanisms that clearly and positively link behavior (exercise) with the exerciser role identity (Dillman, 2007). Exercise identity can fully account for the relationship between competence and prospectively measured exercise behavior, even when controlling for baseline exercise activity. Furthermore, exercise identity could be considered the most prominent prospective correlate of exercise behavior, even after adjusting for exercise activity (Wierts et al., 2024).

Regarding psychological factors, several elements have been proposed to explain the positive effects of exercise on the self, including increased perceived competence related to the physical self and body, enhanced self-acceptance and body satisfaction, a greater sense of autonomy and control, and the incorporation of exercise as a salient aspect of one’s identity. This, in turn, influences the development of exercise-related schemas and subsequently affects information processing (Lindwall, 2004). Exercise identity can mediate the relationship between the exercise environment and adolescent exercise behavior (Su et al., 2024).

Exercise identity is expected to be closely associated with exercise behavior and, in turn, predicted by it. Therefore, the validity of the ‘Exercise Identity Scale’ was examined by assessing exercise-related behaviors among Turkish participants. In light of this information, the hypotheses of the study are as follows:

*H1*: The original “Exercise Identity Measurement Scale” is suitable for Turkish individuals.

*H2*: The original factor structure of the “Exercise Identity Measurement Scale” is suitable for the Turkish version.

## 2 Materials and methods

### 2.1 Participants

A total of 689 individuals participated voluntarily, with 409 (215 male and 194 female individuals) contributing to the exploratory factor analysis to identify the factor structure and 280 (140 male and 140 female individuals) participating in the confirmatory factor analysis to validate the identified structure. All participants were 18 years of age or older. No additional inclusion or exclusion criteria were applied. Table 1 provides a breakdown of the demographic characteristics of the participant groups involved in the study.

**Table 1:** Demographic distributions of EFA and CFA groups.

Sex	EFA		CFA	
	*n*	%	*n*	%
Male	215	52.6	140	50
Female	194	47.4	140	50
Total	409	100	280	100

### 2.2 Instrument

The original Exercise Identity Measurement Scale (EIMS), consisting of nine items (Anderson and Cychosz, 1994) and 12 demographic questions, was also used in the study. In total, three physical education lecturers with advanced proficiency in English translated the EIMS items into Turkish. The translations were then reviewed by an English teacher, who selected the most accurate versions of each item. Subsequently, three additional English teachers conducted a back-translation. All translations were compared with the original scale, and the final Turkish version was completed according to the reconciliation procedures of a total of seven experts. Due to the small number of items on the scale and the simplicity and clarity of the expressions, a pilot group was not required to complete the scale.

The original scale showed a Cronbach’s alpha coefficient of 0.94. All items were significantly intercorrelated, with item–total correlations ranging from 0.87 to 0.55 (mean = 0.77). The original scale was unidimensional, with a single factor having an eigenvalue of 6.09, accounting for 67.6% of the total variance. Factor loadings ranged from 0.91 to 0.62 (Anderson and Cychosz, 1994).

Items of the original EIMS:

1. I consider myself an exerciser.
2. When I describe myself to others, I usually include my involvement in exercise.
3. I have numerous goals related to exercising.
4. Physical exercise is a central factor in my self-concept.
5. I need to exercise to feel good about myself.
6. Others see me as someone who exercises regularly.
7. For me, being an exerciser means more than just exercising.
8. I would feel a real loss if I were forced to give up exercising.
9. Exercising is something I think about often.

The researcher administered the Turkish version of the EIMS using a 7-point Likert scale to participants at the start of the undergraduate theoretical courses. All necessary permissions were obtained from the institution’s administration, and the researchers voluntarily applied the data collection instrument to the students. All participants were encouraged to provide truthful responses.

### 2.3 Data analysis

The study involved utilizing the original nine-item unidimensional scale structure and subsequently testing an eight-item unidimensional model. All analyses were conducted using SPSS 20.0 (IBM Corp, 2011) and AMOS (Arbuckle, 2013). The internal consistency of the scales was evaluated using Cronbach’s alpha coefficients (Cronbach, 1951). The internal consistency of the eight-item unidimensional version was calculated.

Prior to conducting EFA, the data were subjected to assumption testing. Descriptive statistical analyses of responses to the 30-item candidate scale confirmed the absence of missing data. Item-level normality was assessed by examining skewness and kurtosis values. Skewness ranged from −0.018 to 1.077, and kurtosis ranged from −1.394 to −0.278, falling within the acceptable limits of −3.3 to +3.3 for skewness and −7 to +7 for kurtosis, as recommended by Bernstein (2000). These results indicate that the data conformed to a normal distribution.

CFA was used to assess model fit, with indices including CMIN, CMIN/DF, root mean square error of approximation (RMSEA), RMR, SRMR, comparative fit index (CFI), goodness of fit index (GFI), adjusted goodness of fit index (AGFI), normed fit index (NFI), and NNFI. The following benchmarks guided interpretation: CMIN/DF < 3 (Kline, 2016), and RMSEA values between 0.08 and 0.10 for acceptable fit (MacCallum et al., 1996). Traditionally, a threshold of 0.90 is recommended (Shevlin and Miles, 1998). Initially, for CFI, a threshold value of 0.90 was considered acceptable; however, later research suggested that values of 0.95 indicate good fit (Bentler and Bonett, 1980). GFI values between 0 and 1 are considered acceptable. The acceptable level of fit for AGFI is >0.95 (Schumacker and Lomax, 2010). NFI values range from 0 to 1, with a value of 0.90 considered good. For NNFI, values greater than 0.95 are typically indicative of good fit (Hu and Bentler, 1999).

## 3 Results

The factor structure of the Exercise Identity Measurement Scale–Turkish Version was determined using multiple criteria, including the scree plot, total variance explained, and eigenvalues. Item assignment to factors was guided by communalities, explained common variance, factor loadings, and the absence of cross-loadings. To assess the reliability and validity of the Turkish version of the EIMS, the initial nine-item unidimensional structure was examined. After the analysis, one item (X7) was removed from the scale because of a low factor loading (0.396), reducing the number of items to eight. The unidimensional structure was maintained. The internal consistency of the Exercise Identity Measurement Scale–Turkish Version (eight-item unidimensional structure) is presented in Table 2.

**Table 2:** Internal consistency of the EIMS–Turkish version.

Reliability statistics	
Cronbach’s alpha	N. of items
0.941	8

Cronbach’s alpha coefficients for the eight-item unidimensional structure of the scale were satisfactory (*α*=0.941). Table 3 shows the Kaiser–Meyer–Olkin (KMO) data for the Exercise Identity Measurement Scale–Turkish Version.

**Table 3:** KMO data and Bartlett’s test for the EIMS–Turkish version.

KMO and Bartlett’s test		
KMO measure of sampling adequacy.		0.931
Bartlett’s test of sphericity	Approx. chi-square	2732.92
	Df	28
	Sig.	0.000

To determine the suitability of the data derived from the study sample for factor analysis, the KMO value was 0.89, and Bartlett’s test of sphericity was significant. Factor loadings for the Exercise Identity Measurement Scale–Turkish version (eight-item unidimensional structure) are shown in Table 4.

**Table 4:** Factor loadings of the EIMS–Turkish version.

Item no.	Items	Factor loading
1	I consider myself an exerciser	0.822
2	When I describe myself to others, I usually include my involvement in exercise	0.850
3	I have numerous goals related to exercising	0.829
4	Physical exercise is a central factor in my self-concept	0.906
5	I need to exercise to feel good about myself	0.696
6	Others see me as someone who exercises regularly	0.855
7	I would feel a real loss if I were forced to give up exercising	0.796
8	Exercising is something I think about often	0.773
	Eigenvalue	5.68
	Total variance explained (%)	71.00

Extraction Method: Maximum Likelihood.

One factor was extracted; four iterations were required.

**Figure 1:**
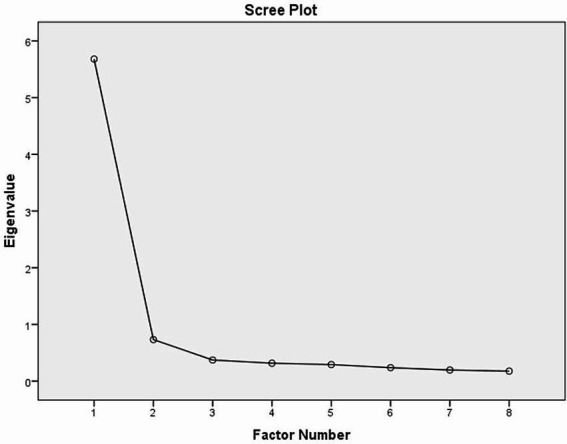
Scree plot of the EIMS–Turkish version.

The factor loadings of the eight items ranged between 0.696 and 0.906. The scree plot from the analysis is presented in Figure 1.

Within the scope of the CFA, fit indices—including chi-square goodness of fit (χ^2^), chi-square/degree of freedom (χ^2^/df), root mean square error of approximation (RMSEA), comparative fit index (CFI), goodness of fit index (GFI), adjusted goodness of fit index (AGFI), and normed fit index (NFI)—are presented in Table 5.

**Table 5:** CFA results of the EIMS–Turkish version.

CMIN	DF	CMIN/DF	RMSEA	RMR	SRMR	CFI	GFI	AGFI	NFI	NNFI
71.12	20	3.556	0.096	0.168	0.0512	0.937	0.933	0.879	0.915	0.911

**Figure 2:**
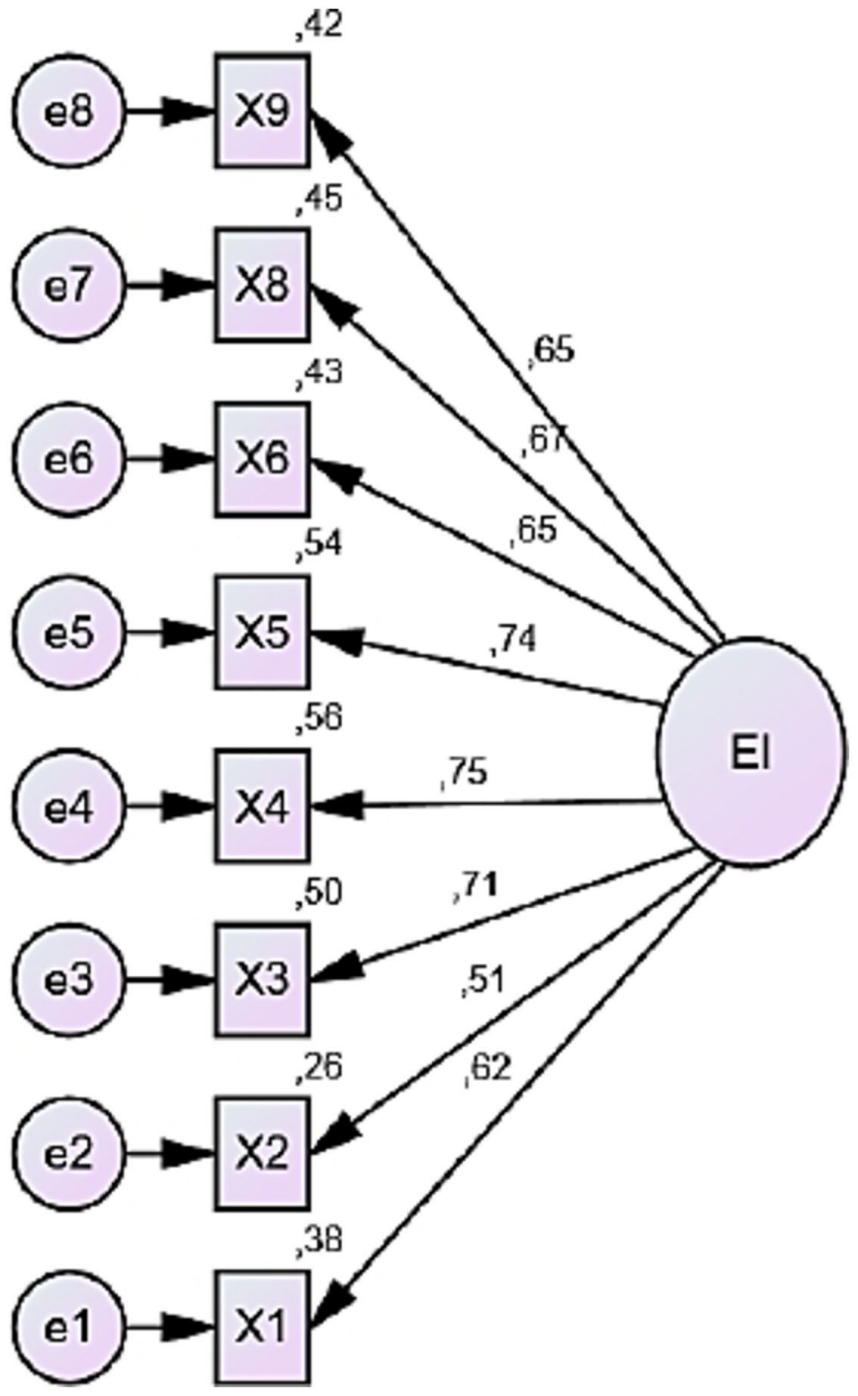
Path diagram of the EIMS–Turkish version.

The path diagram presented in Figure 2 visually represents the CFA model, which was tested to validate the unidimensional structure previously identified through EFA.

## 4 Discussion

This study examined the psychometric properties of the Exercise Identity Measurement Scale among Turkish participants, including construct validity, criterion-related validity, internal consistency, and reliability. In the EFA conducted to assess construct validity, it was observed that the factor loading of the seventh item was 0.304, and this item was removed from the scale due to its very low factor loading. Şenca (2005) stated that “A low factor loading of an item indicates that the item is not strongly associated with the factor in question.” According to the literature, the minimum acceptable factor loading for an item is generally considered to be 0.30, although some theorists argue that it should be 0.40.

Previous studies, such as Anderson and Cychosz (1994), Vlachopoulos et al. (2008), Modrono et al. (2010), Ennigkeit and Hänsel (2018), and Brown and Meca (2025), have shown that the unidimensional model is appropriate for use in Turkish culture. Conversely, Wilson and Muon (2008) found that confirmatory factor analyses indicated that a two-factor model of the Exercise Identity Measurement Scale (EIMS), consisting of role identity and exercise beliefs factors, provided a better fit to the data compared to a unidimensional model. In addition, they reported that correlational and multiple regression analyses suggested that role identity and exercise beliefs were associated with more frequent exercise behavior and greater psychological need satisfaction in exercise. However, this trend was more pronounced for role identity. These findings suggest that the EIMS has the potential to enhance our understanding of exercise identity and underscore the need for further research on the construct validity of the scale.

In another study, Vlachopoulos et al. (2011) found that the factor structure of the EIMS responses was best represented by a two-factor model rather than a unidimensional model. In addition, Murray et al. (2013) used a two-factor EIMS model; however, at the end of the study, they noted that “the two factors were strongly correlated, suggesting a high level of redundancy with one another. Given the high correlation between the two factors, it is possible that the construct is truly unidimensional.”

This study aimed to assess the reliability and validity of the “Exercise Identity Measurement Scale” for a Turkish sample. The results indicated that the eight-item unidimensional structure of the original scale is appropriate for Turkish individuals. Future research could conduct comparative examinations of how this scale functions across different cultures.

## Data Availability

The original contributions presented in the study are included in the article/supplementary material, further inquiries can be directed to the corresponding author.
